# Ethical Leadership, Organisational Justice and Nurses’ Resilience: A Cross‐Sectional Study in Iran

**DOI:** 10.1155/jonm/3166081

**Published:** 2026-10-12

**Authors:** Rafat Rezapour-Nasrabad

**Affiliations:** ^1^ Department of Psychiatric Nursing and Management School of Nursing and Midwifery Shahid Beheshti University of Medical Sciences Tehran Iran sbmu.ac.ir

**Keywords:** ethical leadership, Job Demands–Resources model, nursing management, nursing workforce, organisational justice, resilience, workforce sustainability

## Abstract

**Aim:**

To investigate the relationships among ethical leadership, organisational justice and nurses’ resilience and to determine the extent to which these organisational resources are independently associated with nurses’ resilience within the resource–outcome perspective of the Job Demands–Resources (JD–R) framework.

**Background:**

Developing a resilient nursing workforce has become a strategic priority for healthcare organisations facing persistent nursing shortages, increasing clinical complexity and escalating job demands. Although ethical leadership and organisational justice have each been linked to positive workforce outcomes, their integrated contribution to nurses’ resilience remains insufficiently understood, particularly within nursing management research and in resource‐constrained healthcare systems.

**Methods:**

A cross‐sectional correlational study was conducted among 226 registered nurses employed in teaching hospitals affiliated with Shahid Beheshti University of Medical Sciences, Iran. Participants were recruited using proportional stratified random sampling. Data were collected using validated measures of ethical leadership, organisational justice and resilience. Descriptive statistics, Pearson’s correlation coefficients, and multiple linear regression analyses were performed using SPSS Version 27.

**Results:**

Ethical leadership demonstrated significant positive associations with organisational justice and nurses’ resilience. Organisational justice was also positively associated with resilience and remained a significant independent associate after adjustment for demographic characteristics. Regression analyses indicated that ethical leadership and organisational justice jointly explained significant variance in key resilience dimensions, particularly personal competence, positive acceptance of change and perceived control. Among the organisational factors examined, leadership integrity and distributive justice showed the largest observed correlations with resilience.

**Conclusions:**

This study extends the application of the JD–R model by demonstrating that ethical leadership and organisational justice function as complementary organisational resources that are positively associated with nurses’ resilience. The findings suggest that individual resilience is associated with effective leadership, though it should not be conflated with organisational resilience.

**Implications for Nursing Management:**

Supporting workforce resilience requires organisational strategies that extend beyond individual resilience training. Nurse managers should prioritise ethical leadership development, transparent decision‐making, equitable resource allocation and organisational justice to foster psychologically healthy work environments. Investing in these organisational resources may be associated with nurse well‐being and warrants further interventional research to improve workforce retention and engagement, strengthen organisational performance and ultimately support the delivery of safe, high‐quality and sustainable nursing care.

## 1. Background

The nursing workforce constitutes nearly half of the global health workforce and is fundamental to ensuring the accessibility, continuity, quality and safety of healthcare services worldwide [1]. Nevertheless, healthcare systems continue to face an unprecedented nursing workforce crisis characterised by persistent staff shortages, rising turnover intention, workforce ageing and increasing psychological demands [2, 3]. According to the World Health Organisation (WHO), the shortage of nurses remains one of the greatest barriers to achieving Universal Health Coverage and the Sustainable Development Goals, particularly in low‐ and middle‐income countries where healthcare resources are limited [1, 4, 5].

Although the acute phase of the COVID‐19 pandemic has ended, its organisational and psychological consequences continue to influence nursing practice. Nurses remain exposed to heavy workloads, emotional exhaustion, moral distress, compassion fatigue and burnout, highlighting that workforce sustainability requires more than increasing staffing levels [4, 6–11]. Contemporary healthcare environments are characterised by increasing patient complexity, rapid technological advances, administrative responsibilities, ethical challenges and continuous organisational change, all of which intensify job demands placed on nurses and other healthcare professionals [12–14].

Consequently, resilience has become a strategic priority in nursing management. Resilience is generally defined as the dynamic capacity to adapt positively to adversity, recover from stressful experiences and maintain effective professional functioning under challenging circumstances [15–17]. Increasing evidence indicates that resilient nurses report lower burnout and turnover intention, greater job satisfaction and work engagement and provide higher‐quality patient care [18–21]. Importantly, resilience is now recognised as a dynamic outcome that is associated with organisational factors that can be strengthened through supportive workplace environments rather than merely as an individual personality trait [17, 21, 22].

The Job Demands–Resources (JD–R) model provides one of the most comprehensive theoretical frameworks for understanding how workplace conditions influence employee well‐being and organisational outcomes [3, 8]. According to this model, every occupation involves a combination of job demands, which require sustained physical or psychological effort, and job resources, which help employees achieve work goals, act as resource–outcome pathways, and promote motivation and psychological well‐being [10, 11].

Within nursing, excessive workload, staff shortages, emotional labour, shift work, ethical dilemmas and increasing administrative responsibilities represent major job demands that contribute to stress, burnout and turnover intention [23–25]. In contrast, organisational resources including supportive leadership, fair management practices, teamwork, professional autonomy and psychological safety enhance employee motivation, work engagement, resilience and organisational commitment by acting as complementary resources associated with positive outcomes in demanding work environments [9, 11, 26].

Ethical leadership represents one of the most influential organisational resources within the JD–R framework [27]. Ethical leaders promote integrity, transparency, fairness, accountability and respectful interpersonal relationships while encouraging employee participation in decision‐making and ethical practice [28–30]. Previous studies have consistently demonstrated that ethical leadership is associated with higher job satisfaction, stronger organisational commitment, greater psychological empowerment, lower burnout, improved teamwork and enhanced patient safety culture among nurses [31–34]. From the JD–R perspective, ethical leadership strengthens employees’ psychological resources by fostering trust, optimism, self‐efficacy and psychological safety, thereby enhancing their ability to cope with occupational stressors [11, 35–37].

Another key organisational resource is organisational justice, which refers to employees’ perceptions of fairness regarding organisational procedures, resource allocation, interpersonal treatment and managerial communication [38, 39]. Nurses who perceive higher organisational justice report greater job satisfaction, organisational commitment, work engagement and psychological well‐being, whereas perceptions of injustice have been associated with burnout, moral distress, absenteeism and turnover intention [27, 37, 40–45]. Within the JD–R framework, organisational justice reduces uncertainty, strengthens employees’ sense of control and trust and facilitates the development of resilience by providing a fair and supportive organisational environment [11, 43, 45].

Although ethical leadership, organisational justice and resilience have each received considerable attention in nursing research, important gaps remain. Previous studies have primarily examined ethical leadership and organisational justice separately, focusing on outcomes such as burnout, job satisfaction, or organisational commitment rather than resilience [36, 43, 44]. Likewise, resilience has often been investigated from an individual perspective, emphasising personality traits, coping strategies, emotional intelligence, or psychological capital while paying relatively little attention to organisational determinants that may strengthen nurses’ adaptive capacity [27, 40].

Furthermore, relatively few studies have integrated ethical leadership and organisational justice within a single theoretical framework capable of explaining how organisational resources jointly influence nurses’ resilience [41]. Evidence from Middle Eastern healthcare systems, particularly Iran, also remains limited despite increasing workforce shortages, growing service demands and persistent organisational challenges [42]. Addressing these gaps is important because resilience is increasingly recognised as an outcome associated with organisational factors rather than solely an individual characteristic, requiring supportive leadership and equitable management practices to ensure workforce sustainability [31, 43, 45].

Guided by the JD–R model, the present study conceptualises ethical leadership and organisational justice as complementary organisational resources that may be associated with nurses’ resilience by promoting positive psychological adaptation (resource–outcome pathway) [37, 38]. This framework provides a theoretically coherent explanation for understanding how leadership behaviours and perceptions of fairness contribute to workforce resilience within hospital settings.

This study aimed to investigate the relationships among ethical leadership, organisational justice and resilience among nurses working in Iranian teaching hospitals. Specifically, the study examined whether ethical leadership and organisational justice were associated with nurses’ resilience and whether these organisational resources were independently associated with resilience after controlling for their shared effects.

Based on the JD–R model and previous empirical evidence, the following hypotheses were proposed:

• H1: Ethical leadership is positively associated with nurses’ resilience.
• H2: Organisational justice is positively associated with nurses’ resilience.
• H3: Ethical leadership is positively associated with organisational justice.
• H4: Ethical leadership and organisational justice are independently associated with nurses’ resilience.

By examining these hypotheses, this study extends current knowledge regarding the organisational determinants of resilience and provides evidence to support leadership development and organisational policies aimed at strengthening resilient nursing workforces. The findings are expected to inform nursing managers and healthcare policymakers seeking to create healthy, equitable and sustainable work environments that enhance both staff well‐being and quality of patient care.

## 2. Methods and Materials

### 2.1. Study Design and Setting

This cross‐sectional correlational study was conducted between June and September 2025 among registered nurses working in teaching hospitals affiliated with Shahid Beheshti University of Medical Sciences, Tehran, Iran. The study was guided by the JD–R model, which conceptualises ethical leadership and organisational justice as organisational resources that are associated with nurses’ resilience in demanding healthcare environments. The study adhered to the Strengthening the Reporting of Observational Studies in Epidemiology (STROBE) guidelines for reporting observational studies.

### 2.2. Participants and Sampling

The study population comprised all registered nurses employed in teaching hospitals affiliated with Shahid Beheshti University of Medical Sciences during the study period. Nurses were eligible to participate if they

• held at least a bachelor’s degree in nursing;
• had a minimum of one year of clinical work experience;
• were employed in direct patient care;
• agreed to participate voluntarily by providing written informed consent.

Nurses who were on extended leave during the data collection period, occupied exclusively administrative positions, or submitted incomplete questionnaires were excluded from the study.

A total of 226 nurses meeting the eligibility criteria participated in the study. Participants were selected using a proportional stratified random sampling technique. To ensure a representative sample, we first determined the total number of eligible nurses employed in each of the six affiliated teaching hospitals. The target sample size (*N* = 226) was then proportionally allocated across these six strata (hospitals) based on the percentage of the total nursing workforce each hospital contributed. For instance, larger hospitals with a greater number of eligible nurses provided a proportionally larger share of the final sample. The total number of eligible nurses was 1200. Proportional allocation resulted in stratum sizes of 260, 250, 250, 220, 130 and 90 across the six hospitals. Of the 281 questionnaires distributed, 15 were incomplete, and 40 nurses refused (response rate = 85%). A nonresponse bias analysis comparing early and late respondents showed no significant differences, suggesting minimal nonresponse bias. This approach ensured that the demographic and organisational characteristics of the final sample accurately reflected the broader nursing population across all affiliated hospitals.

### 2.3. Sample Size

The sample size was determined using G∗Power 3.1 for multiple linear regression. Assuming a medium effect size (*f* ^2^ = 0.15), an alpha of 0.05, a power of 0.80 and 8 predictors, the required sample was 109. Accounting for a 20% attrition rate and to ensure sufficient power for dimension‐level exploratory analyses, the target sample size was increased to 226, which exceeds the rule of thumb of 15 cases per predictor.

### 2.4. Data Collection Instruments

Data were collected using four standardised questionnaires.

### 2.5. Demographic Characteristics Questionnaire

Participants completed a demographic questionnaire including age, gender, marital status, educational level, employment status, work experience, hospital ward, work shift and other relevant occupational characteristics.

### 2.6. Ethical Leadership Questionnaire

Ethical leadership was assessed using the Ethical Leadership at Work Questionnaire (ELW), originally developed by Kalshoven et al. [46]. In the present study, the validated 23‐item version comprising four dimensions (integrity, power sharing, ethical guidance and fairness), as reported by Zarei Matin et al. [47], was used. Responses were rated on a five‐point Likert scale ranging from 1 (*strongly disagree*) to 5 (*strongly agree*), with higher scores indicating higher perceived ethical leadership. The Persian version of the questionnaire has demonstrated acceptable psychometric properties in previous studies.

### 2.7. Organisational Justice Questionnaire

Perceived organisational justice was measured using the Niehoff and Moorman Organisational Justice Questionnaire [48], comprising 20 items evaluating employees’ perceptions of fairness within the organisation. Items were scored using a five‐point Likert scale, with higher scores representing greater perceived organisational justice. The questionnaire has been widely used in nursing research and has demonstrated satisfactory validity and reliability in Iranian populations.

### 2.8. Resilience Questionnaire

Nurses’ resilience was measured using the Connor–Davidson Resilience Scale (CD‐RISC‐25), developed by Connor and Davidson [49], consisting of 25 items assessing the individual’s ability to adapt successfully to adversity. Each item was scored on a five‐point Likert scale, producing a total score ranging from 0 to 100, with higher scores indicating greater resilience. The Persian version of the CD‐RISC has shown good validity and internal consistency in previous studies.

### 2.9. Data Collection Procedure

Following approval from the university ethics committee and permission from hospital administrators, eligible nurses were invited to participate in the study. The objectives of the study were explained to all participants, and written informed consent was obtained before data collection. Participants completed the questionnaires anonymously during their work shifts at a convenient time. The completed questionnaires were collected in sealed envelopes to ensure confidentiality and minimise response bias.

### 2.10. Ethical Considerations

The study was approved by the Ethics Committee of Shahid Beheshti University of Medical Sciences (IR.SBMU.PHARMACY.REC.1404.031). Participation was entirely voluntary, and participants were informed of their right to withdraw from the study at any stage without any consequences. All collected data were treated confidentially and analysed anonymously.

### 2.11. Statistical Analysis

Data were analysed using SPSS Statistics Version 27. Descriptive statistics, including frequencies, percentages, means and standard deviations, were used to summarise participants’ characteristics and study variables.

The normality of continuous variables was assessed before inferential analyses. Pearson’s correlation coefficient was applied to examine the relationships among ethical leadership, organisational justice and resilience. Multiple linear regression analysis was subsequently performed to determine whether ethical leadership and organisational justice were independently associated with nurses’ resilience while controlling for demographic characteristics. Statistical significance was established at *p* < 0.05.

Because participants were recruited from six teaching hospitals, potential clustering effects were considered. We calculated the intraclass correlation coefficient (ICC) for the total resilience score across the six hospitals to assess data dependency. The ICC was 0.018, indicating that only 1.8% of the variance in resilience was attributable to between‐hospital clustering. Given this negligible ICC and the small number of level‐2 clusters (*N* = 6), which can lead to unstable variance estimates in multilevel models, ordinary multiple linear regression was deemed appropriate, as the assumption of independent observations was sufficiently met. Additionally, bivariate Pearson correlations were calculated between the subscales of ethical leadership/organisational justice and the total resilience score to generate Figure 1. No adjustment for multiple comparisons was applied for these exploratory subscale analyses.

**FIGURE 1:**
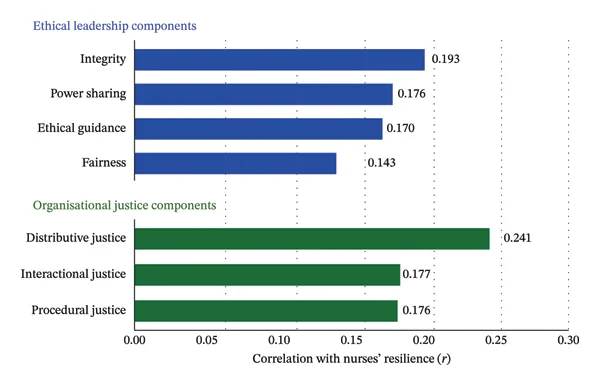
Ranking of organisational predictors according to their correlation with nurses’ resilience.

To assess common method bias, Harman’s single‐factor test was conducted. The unrotated first factor accounted for 28.4% of the variance, which is below the 50% threshold, indicating that common method bias was not a pervasive issue.

## 3. Results

The results of the data analysis are summarised in the following tables.

### 3.1. Participant Characteristics

According to Table 1, the majority of the participants were female (69.9%), married (65.9%), and held a bachelor’s degree in nursing (85.8%). The largest proportion of nurses were under 30 years of age (39.8%). Regarding employment status, most participants were employed on an official/permanent basis (35.8%). The nurses were distributed across six teaching hospitals, with the highest representation from Tajrish (21.7%), Loghman (20.8%) and Imam Hossein (20.8%) hospitals. Clinically, more than half of the sample worked in internal medicine wards (58.4%), followed by surgical wards (41.6%).

**TABLE 1:** Demographic and occupational characteristics of the participating nurses (*N* = 226).

Variable	Category	*n* (%)
Gender	Female	158 (69.9)
	Male	68 (30.1)

Age (years)	< 30	90 (39.8)
	31–40	80 (35.4)
	41–50	43 (19.0)
	> 50	13 (5.8)

Marital status	Married	149 (65.9)
	Single	77 (34.1)

Educational level	Bachelor’s degree	194 (85.8)
	Master’s degree	32 (14.2)

Employment status^∗^	Official (permanent)	81 (35.8)
	Contractual (fixed‐term)	70 (31.0)
	Project‐based	40 (17.7)
	Temporary contract	35 (15.5)

Hospital	Tajrish	49 (21.7)
	Loghman	47 (20.8)
	Imam Hossein	47 (20.8)
	Taleghani	41 (18.1)
	Modarres	24 (10.6)
	Labbafinejad	18 (8.0)

Working ward	Internal medicine	132 (58.4)
	Surgical	94 (41.6)

^∗^Employment categories reflect the standard nursing contractual frameworks in Iran.

Based on Table 2, a total of 226 registered nurses participated in this study. Participants were recruited from teaching hospitals affiliated with Shahid Beheshti University of Medical Sciences. Among the demographic variables examined, only marital status was significantly associated with nurses’ resilience (*p* = 0.040), whereas gender, educational level, age, work experience and employment status showed no statistically significant associations.

**TABLE 2:** Association between demographic characteristics and resilience (*n* = 226).

Variable	Statistical test	Test statistic	*p* value
Gender	Independent *t*‐test	*t* = −0.64	0.521
Educational level	Independent *t*‐test	*t* = −0.30	0.762
Marital status	Independent *t*‐test	*t* = −2.07	0.040
Age	One‐way ANOVA	*F* = 0.91	0.437
Work experience	One‐way ANOVA	*F* = 0.95	0.390
Employment status	One‐way ANOVA	*F* = 0.33	0.800

Cronbach’s *α* values represent internal consistency reliability. CFA fit indices demonstrate the construct validity of the measurement models within the present study sample (see Table 3).

**TABLE 3:** Psychometric properties of the study instruments (*N* = 226).

Instrument	Items	Cronbach’s *α*	*χ* ^2^/df	CFI	TLI	RMSEA (90% CI)
Ethical Leadership at Work Questionnaire (ELW)	23	0.87	2.45	0.92	0.91	0.06 (0.04–0.08)
Niehoff and Moorman Organisational Justice Questionnaire	20	0.89	2.10	0.94	0.93	0.05 (0.03–0.07)
Connor–Davidson Resilience Scale (CD‐RISC‐25)	25	0.91	2.85	0.90	0.89	0.07 (0.05–0.09)

*Note:* *χ* ^2^/df = chi‐square to degrees of freedom ratio.

Abbreviations: CFA, confirmatory factor analysis; CFI, comparative fit index; CI, confidence interval; RMSEA, root mean square error of approximation; TLI, Tucker–Lewis index.

### 3.2. Correlations Between Ethical Leadership, Organisational Justice and Resilience Dimensions

According to Table 4, Pearson correlation analysis demonstrated that personal competence had a significant positive association with both ethical leadership (*r* = 0.210, *p* < 0.001) and organisational justice (*r* = 0.250, *p* < 0.001). Similarly, positive acceptance of change and secure relationships were positively correlated with ethical leadership (*r* = 0.180, *p* = 0.006) and organisational justice (*r* = 0.195, *p* = 0.003). Additionally, the control dimension showed significant positive correlations with ethical leadership (*r* = 0.150, *p* = 0.024) and organisational justice (*r* = 0.170, *p* = 0.010). In contrast, the dimensions of trust in individual instincts and tolerance of negative affect and spiritual influences showed no statistically significant associations with either ethical leadership or organisational justice (*p* > 0.05 for all).

**TABLE 4:** Pearson correlations between resilience dimensions and organisational variables (*N* = 226).

Resilience dimension	Ethical leadership		Organisational justice	
	*r*	*p* value	*r*	*p* value
Personal competence	0.210	< 0.001	0.250	< 0.001
Trust in individual instincts and tolerance of negative affect	0.085	0.210	0.092	0.175
Positive acceptance of change and secure relationships	0.180	0.006	0.195	0.003
Control	0.150	0.024	0.170	0.010
Spiritual influences	0.045	0.500	0.060	0.380

### 3.3. Correlations Between Leadership and Justice Dimensions With Resilience

Based on Table 5, all dimensions of ethical leadership and organisational justice were positively associated with nurses’ resilience. Among the ethical leadership dimensions, integrity demonstrated the largest observed correlation with resilience (*r* = 0.193, *p* = 0.004), whereas fairness showed the weakest association (*r* = 0.143, *p* = 0.031). Among the organisational justice dimensions, distributive justice had the largest observed relationship with resilience (*r* = 0.241, *p* < 0.001).

**TABLE 5:** Pearson correlations between ethical leadership dimensions, organisational justice dimensions and resilience.

Predictor	*r*	*p* value
Ethical leadership		
Integrity	0.193	0.004
Power sharing	0.176	0.008
Ethical guidance	0.170	0.010
Fairness	0.143	0.031
Organisational justice		
Distributive justice	0.241	< 0.001
Procedural justice	0.176	0.008
Interactional justice	0.177	0.008

### 3.4. Multiple Regression Analysis

A multiple regression analysis for the total resilience score was conducted to fully test H4, which showed that organisational justice was a significant independent associate, while ethical leadership showed a borderline association (*p* = 0.051). According to Table 6, multiple regression analyses were conducted to evaluate the independent contributions of ethical leadership and organisational justice to the variance in the significant dimensions of resilience. For personal competence, the model explained 12% of the variance (*R* ^2^ = 0.120), with both ethical leadership (*β* = 0.148, *p* = 0.031) and organisational justice (*β* = 0.214, *p* = 0.001) serving as significant independent contributors. Whereas the independent effect of ethical leadership on control did not reach statistical significance (*p* = 0.065), suggesting it may not independently link to this specific dimension.

**TABLE 6:** Multiple regression analyses for total resilience score and its significant dimensions (*N* = 226).

Resilience dimension (dependent variable)	Predictor	*B*	SE	*β*	95% CI for *B*	*p* value	Model *R* ^2^	Model *F*	Model *p*
Total resilience score	(Constant)	57.1	4.50	—	48.23 to 65.97	< 0.001	0.150	19.70	< 0.001
	Ethical leadership	0.780	0.398	0.145	−0.004 to 1.564	0.051	0.120	15.24	< 0.001
	Organisational justice	1.480	0.500	0.225	0.500 to 2.460	0.003	0.060	7.06	< 0.001

Personal competence	(Constant)	42.15	4.320	—	33.73 to 50.57	< 0.001	0.120	15.24	< 0.001
	Ethical leadership	0.142	0.065	0.148	0.013 to 0.271	0.031			
	Organisational justice	0.265	0.082	0.214	0.103 to 0.427	0.001			

Positive acceptance of change	(Constant)	15.20	1.850	—	11.55 to 18.85	< 0.001	0.060	7.06	< 0.001
	Ethical leadership	0.085	0.042	0.140	0.002 to 0.168	0.045			
	Organisational justice	0.150	0.055	0.195	0.041 to 0.259	0.007			

Control	(Constant)	8.50	1.120	—	6.29 to 10.71	< 0.001	0.032	3.66	0.027
	Ethical leadership	0.065	0.035	0.130	−0.004 to 0.134	0.065			
	Organisational justice	0.110	0.045	0.185	0.021 to 0.199	0.016			

As shown in Table 7, all hypothesised relationships were supported by the study findings. Pearson correlation analyses demonstrated significant positive associations among ethical leadership, organisational justice and nurses’ resilience. In addition, multiple regression analysis indicated that both ethical leadership and organisational justice were significant independently associated with resilience. These findings provide empirical support for the proposed conceptual framework and suggest that organisational resources play a meaningful role in enhancing nurses’ resilience.

**TABLE 7:** Summary of hypothesis testing.

Hypothesis	Relationship	Statistical test	Result
H1	Ethical leadership ⟶ resilience	Pearson correlation	Supported
H2	Organisational justice ⟶ resilience	Pearson correlation	Supported
H3	Ethical leadership ⟶ organisational justice	Pearson correlation	Supported
H4	Ethical leadership and organisational justice are independently associated with resilience	Multiple regression	Supported

According to Table 8, ethical leadership and organisational justice contributed differently across the resilience dimensions. The strongest independent contribution was observed for personal competence, followed by positive acceptance of change, secure relationships and control. Together, the organisational variables explained 12.0%, 6.0% and 3.2% of the variance in these dimensions, respectively. However, no significant independent contributions were found for trust in individual instincts and tolerance of negative affect or spiritual influences, suggesting that these aspects of resilience may be influenced by factors beyond the organisational resources assessed in the present study.

**TABLE 8:** Contribution of organisational variables to resilience dimensions.

Resilience dimension	Significant	Variance explained (%)
Personal competence	Yes	12
Positive acceptance	Yes	6
Control	Yes	3.2
Trust in instincts	No	—
Spiritual influences	No	—

Figure 2 presents the JD–R‐informed conceptual framework examining the associations among ethical leadership, organisational justice and nurses’ resilience. H1 to H3 represent the study hypotheses tested via bivariate and multivariable analyses.

**FIGURE 2:**
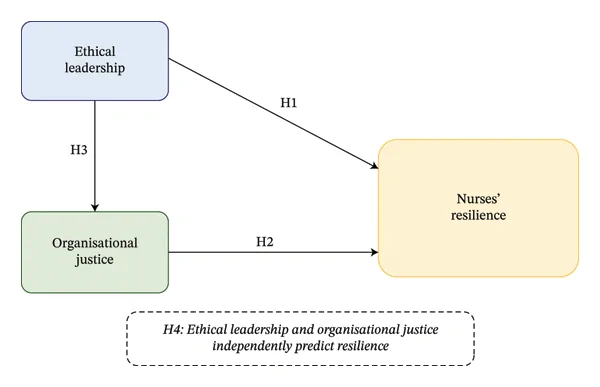
Conceptual framework of organisational resources and nurses’ resilience (JD–R‐informed model).

As shown, ethical leadership was positively associated with both organisational justice and resilience, while organisational justice was also positively related to resilience. All hypothesised relationships were statistically significant and in the expected direction. The model provides empirical support for the JD–R framework by demonstrating that organisational resources contribute to resilience among nurses. These findings highlight the importance of ethical leadership and organisational justice as key workplace resources that may strengthen nurses’ adaptive capacity in demanding healthcare environments.

Figure 1 presents the ranking of the dimensions of ethical leadership and organisational justice according to the strength of their correlations with nurses’ resilience. Among the ethical leadership dimensions, the largest observed positive correlation with resilience (*r* = 0.193), followed by power sharing (*r* = 0.176), ethical guidance (*r* = 0.170) and fairness (*r* = 0.143). Among the dimensions of organisational justice, the largest observed correlation with resilience (*r* = 0.241), followed by interactional justice (*r* = 0.177) and procedural justice (*r* = 0.176). Overall, all dimensions exhibited statistically significant positive correlations with nurses’ resilience (*p* < 0.05), with distributive justice emerging as the dimension with the largest observed bivariate association with resilience.

## 4. Discussion

The present study examined the relationships among ethical leadership, organisational justice and nurses’ resilience within the framework of the JD–R model. Overall, the findings supported all study hypotheses, demonstrating that ethical leadership and organisational justice were positively associated with nurses’ resilience and were independently associated with nurses’ adaptive capacity. Ethical leadership was also positively associated with organisational justice, indicating that supportive leadership behaviours and perceptions of fairness coexist within healthy organisational environments. These findings provide empirical support for a JD–R‐informed resource framework by demonstrating that organisational resources play an important role in promoting resilience among nurses working in demanding healthcare settings [8–11, 38, 45].

The findings reinforce the resource‐orientated proposition of the JD–R framework, suggesting that job resources are associated with positive outcomes while promoting motivation, psychological well‐being and positive organisational outcomes [10, 11]. Nursing is characterised by persistent exposure to heavy workloads, emotional labour, staff shortages, ethical dilemmas and increasing patient complexity [4, 6, 7, 12, 13]. Under such conditions, organisational resources become essential for maintaining nurses’ psychological functioning. The positive associations observed in this study suggest that ethical leadership and organisational justice serve as potentially protective workplace resources that enable nurses to cope more effectively with occupational stressors and maintain professional performance despite challenging working environments.

One of the principal findings was the significant positive relationship between ethical leadership and resilience. Nurses who perceived higher levels of ethical leadership reported greater resilience, with integrity emerging as the leadership dimension with the largest observed correlation with resilience. This finding is consistent with previous nursing research demonstrating that ethical leadership is associated with trust, psychological safety, work engagement, organisational commitment and job satisfaction while reducing burnout and turnover intention [28–36]. Ethical leaders create work environments characterised by transparency, accountability, fairness and respect, thereby linking employees’ confidence in their managers and organisations. Such leadership behaviours provide nurses with psychological resources that facilitate adaptive responses to workplace adversity.

From the JD–R perspective, ethical leadership functions as a critical organisational resource by enhancing self‐efficacy, perceived organisational support, optimism and trust [10, 11, 38]. These psychological resources increase nurses’ capacity to manage stressful clinical situations and recover from occupational challenges. The particularly strong association observed for integrity suggests that consistency between leaders’ values and behaviours is especially important in healthcare organisations where nurses frequently encounter ethical uncertainty and high‐stakes clinical decision‐making. Leaders who consistently demonstrate honesty, fairness and ethical conduct may be associated with nurses’ confidence in organisational processes and create psychologically safe environments that foster resilience.

The present study also identified organisational justice as an important independent associate of resilience. Nurses who perceived greater fairness within their organisations reported higher resilience, and organisational justice remained a significant independent associate after accounting for ethical leadership. Among the justice dimensions, distributive justice demonstrated the largest observed association with resilience, followed by interactional and procedural justice. These findings are consistent with previous evidence showing that perceptions of fairness improve psychological well‐being, work engagement, organisational commitment and job satisfaction while reducing burnout and turnover intention among nurses [27, 38–43].

Within the JD–R framework, organisational justice represents an important job resource because it is associated with employees’ perceptions of predictability, control, trust and organisational support [11]. Fair allocation of workloads, rewards, opportunities and organisational resources reduces uncertainty and psychological strain associated with nurses’ confidence in their work environment. The prominent role of distributive justice observed in this study may reflect the practical realities of hospital nursing, where equitable workload distribution, staffing adequacy, recognition and career opportunities directly influence employees’ daily experiences. In resource‐constrained healthcare systems, perceived inequity may substantially increase occupational stress, whereas equitable organisational practices may reinforce resilience by enhancing nurses’ motivation and sense of professional value.

An important contribution of the present study is its integration of ethical leadership and organisational justice within a single theoretical framework. Previous nursing studies have frequently examined these organisational constructs independently, often focusing on burnout, job satisfaction, or organisational commitment [31, 36, 42]. In contrast, the present study demonstrates that ethical leadership and organisational justice operate as complementary organisational resources that jointly contribute to resilience. This integrated perspective extends the application of a JD–R‐informed resource perspective to nursing management and provides a more comprehensive explanation of how supportive organisational environments are associated with nurses’ adaptive capacity [37, 44, 45].

Another noteworthy finding was that organisational resources contributed differently across resilience dimensions. Ethical leadership and organisational justice were significantly associated with personal competence, positive acceptance of change and control, whereas no significant relationships were observed with trust in individual instincts or spiritual influences. These findings suggest that organisational resources primarily influence resilience dimensions that are closely linked to workplace experiences and interpersonal interactions rather than relatively stable personality traits or individual belief systems. This distinction further supports the conceptualisation of resilience as a multidimensional construct shaped by both personal and organisational factors.

The findings have several important implications for nursing management. First, resilience should be considered an organisational responsibility rather than solely an individual competency. Although resilience training programmes remain valuable, organisational interventions targeting leadership behaviours and workplace fairness are likely to produce broader and more sustainable improvements in nurses’ well‐being. Healthcare organisations should therefore invest in leadership development programmes emphasising ethical decision‐making, integrity, transparent communication, employee participation and accountability. Simultaneously, managers should link distributive, procedural and interactional justice by ensuring equitable workload allocation, transparent promotion systems, fair performance evaluation and respectful communication throughout the organisation.

These organisational strategies may be associated not only with stronger resilience but also to improved nurse retention, greater work engagement, lower burnout and higher quality of patient care. Given the continuing global nursing shortage and increasing complexity of healthcare delivery, ethical leadership and organisational justice may help support resilience, warranting further interventional research for verification for maintaining a resilient nursing workforce [1–3, 20, 21]. The findings therefore have direct relevance for nurse managers, hospital administrators and healthcare policymakers seeking to improve both employee well‐being and organisational performance.

This study has several strengths. It was theoretically grounded in the JD–R model, simultaneously examined two major organisational resources within a single conceptual framework, and employed validated measurement instruments among nurses working in multiple teaching hospitals. Furthermore, due to the cross‐sectional design, these associations should not be interpreted as causal. While the JD–R model posits that resources buffer demands, reverse causation is also plausible; for instance, highly resilient nurses may perceive their leaders and organisational environments more positively. Nevertheless, several limitations should be acknowledged. The cross‐sectional design precludes causal inference, and all variables were measured using self‐report questionnaires, creating the possibility of common method bias. In addition, participants were recruited from teaching hospitals affiliated with a single university, which limits the generalisability of the findings to similar teaching hospitals in Iran.

Future longitudinal and multicentre studies are needed to confirm the causal pathways linking ethical leadership, organisational justice and resilience. Intervention studies evaluating leadership development programmes and organisational justice initiatives would provide stronger evidence regarding effective organisational strategies for enhancing resilience. Future research should also investigate potential mediating mechanisms, including psychological safety, work engagement, burnout, perceived organisational support and psychological capital, as well as moderating variables such as organisational culture, clinical speciality and professional experience. Finally, we conducted multiple exploratory correlations and regressions across resilience dimensions without adjusting for multiple comparisons; thus, these specific dimension‐level findings should be interpreted with caution and confirmed in future confirmatory studies.

In summary, this study extends current knowledge by demonstrating that ethical leadership and organisational justice are complementary organisational resources that contribute to nurses’ resilience within the JD–R framework [3, 27]. The findings highlight that resilience is not solely an individual characteristic but also an organisational capability that can be linked to ethical leadership, equitable management practices and supportive work environments. Investing in these organisational resources may represent an effective strategy for building resilient nursing workforces capable of sustaining high‐quality patient care in increasingly demanding healthcare systems.

## 5. Conclusion

This study demonstrated that ethical leadership and organisational justice are important organisational resources associated with nurses’ resilience in teaching hospitals. Guided by the JD–R model, the findings showed that both ethical leadership and organisational justice were positively related to resilience and independently associated with nurses’ adaptive capacity. Among the organisational justice dimensions, distributive justice exhibited the largest observed association with resilience, while integrity emerged as the component with the largest observed association with ethical leadership. These findings suggest that resilience is shaped not only by individual characteristics but also by the quality of leadership and the fairness of organisational environments. The study extends current nursing management research by integrating ethical leadership and organisational justice within a single theoretical framework to explain nurses’ resilience. This integrated perspective provides empirical support for a JD–R‐informed resource framework and highlights the importance of organisational resources in promoting psychological well‐being and workforce sustainability in demanding healthcare settings.

From a practical perspective, the findings indicate that healthcare organisations should complement individual resilience‐building programmes with organisational interventions that strengthen ethical leadership, promote fair management practices and foster supportive workplace cultures. Investing in leadership development, transparent decision‐making, equitable resource allocation and respectful interpersonal relationships may help support nurses’ resilience, though interventional studies are needed for verification, staff well‐being and the quality and safety of patient care.

As healthcare systems continue to face increasing workforce shortages and escalating clinical demands, developing resilient nursing workforces has become an international priority. The findings of this study suggest that the association of ethical leadership and organisational justice represents a feasible and sustainable organisational strategy for enhancing nurses’ resilience and supporting the long‐term resilience of healthcare organisations. Future longitudinal and intervention‐based studies are recommended to further examine the causal mechanisms linking organisational resources to resilience and to evaluate the effectiveness of leadership and organisational interventions across diverse healthcare settings.

## Author Contributions

Study design, data collection, data analysis, manuscript writing: Rafat Rezapour‐Nasrabad.

## Funding

No funding was received for this research.

## Ethics Statement

This study was approved by the Ethics Committee of Shahid Beheshti University of Medical Sciences (Approval No. IR.SBMU.PHARMACY.REC.1404.031). The study was conducted in accordance with established ethical principles in nursing research and in compliance with the Declaration of Helsinki and its later amendments. Participation was voluntary, and written informed consent was obtained from all participants before data collection. Participants were informed of the study objectives, assured of the confidentiality and anonymity of their data, and informed of their right to withdraw from the study at any time without any consequences.

## Consent

Please see the Ethics Statement.

## Conflicts of Interest

The author declares no conflicts of interest.

## Data Availability

The data that support the findings of this study are available on request from the corresponding author. The data are not publicly available due to privacy or ethical restrictions.
